# Yield Response and Physiological Adaptation of Green Bean to Photovoltaic Greenhouses

**DOI:** 10.3389/fpls.2021.655851

**Published:** 2021-05-24

**Authors:** Marco Cossu, Antonella Sirigu, Paola A. Deligios, Roberta Farci, Gianluca Carboni, Giulia Urracci, Luigi Ledda

**Affiliations:** ^1^Department of Agriculture, University of Sassari, Sassari, Italy; ^2^Agricultural Research Agency of Sardinia (Agris Sardegna), Cagliari, Italy; ^3^Department of Agricultural, Food and Environmental Sciences, Polytechnic University of Marche, Ancona, Italy

**Keywords:** efficiency, energy, French bean, shading, solar radiation, transpiration

## Abstract

The cultivation of the horticultural crops inside photovoltaic greenhouses (PVG) should be studied in relation to the shading cast by the photovoltaic (PV) panels on the roof. This work evaluated the green bean cultivation inside PVGs with a percentage of the greenhouse area covered with PV panels (PV cover ratio, *PV*_*R*_) ranging from 25 to 100%. Three dwarf green bean cycles (*Phaseolus vulgaris* L., cv. Valentino) were conducted inside an iron–plastic PVG with a *PV*_*R*_ of 50%. The average yield was 31% lower than a conventional greenhouse. Adverse effects on quality were noticed under the PV roof, including a reduction of pod weight, size, and caliber. Negative net photosynthetic assimilation rates were observed on the plants under the PV roof, which adapted by relocating more resources to the stems, increasing the specific leaf area (SLA), leaf area ratio (LAR), and the radiation use efficiency (RUE). The fresh yield increased by 0.44% for each additional 1% of cumulated PAR. Based on the linear regressions between measured yield and cumulated PAR, a limited yield reduction of 16% was calculated inside a PVG with maximum *PV*_*R*_ of 25%, whereas an average yield loss of 52% can occur with a *PV*_*R*_ of 100%. The economic trade-off between energy and green bean yield can be achieved with a *PV*_*R*_ of 10%. The same experimental approach can be used as a decision support tool to identify other crops suitable for cultivation inside PVGs and assess the agricultural sustainability of the mixed system.

## Introduction

The European Union (UE) recently approved the “European Green Deal,” an imposing roadmap to fund the EU transition toward a green and circular economy with no net gas emissions by 2050 (European Commission, 2020). The sustainable agriculture, the energy efficiency, and the production of clean energy are the pillars to achieve this objective, which should take into account the economic, environmental, social benefits, and impacts of this transition. The EU countries encouraged the renewable energy sources, among which the photovoltaic (PV) technology, by means of subsidy policies, applied also to agriculture (Tudisca et al., 2013). The ground-based PV systems in agricultural land are not allowed in some southern EU countries such as Italy and France, due to their conflict between land and energy, including land deterioration and speculation behind the public incentives provided for the PV energy production (Poncet et al., 2012; Colantoni et al., 2015). For this reason, the installation of PV systems in agriculture was moved to rural buildings or greenhouses, leading to the spread of the PV greenhouse (PVG), which integrates the PV panels on the roof (Castellano, 2014; Sgroi et al., 2014). The strength of the PVG relies on the combination of energy and food production in a unique built-up structure, contributing to the diversification of the farm income (Ureña-Sánchez et al., 2012; Marucci et al., 2018). In locations with high natural irradiation, such as the Mediterranean regions, the shading is commonly applied through shading nets or whitening to mitigate the excess of temperature (Baille et al., 2001; Ahemd et al., 2016), although reducing the solar radiation decreases both the photosynthesis and productivity of the plants, even in summer (Marcelis et al., 2006). On the other hand, the shading can improve the radiation use efficiency by increasing the fraction of diffuse irradiance (Baudoin et al., 2017). Inside PVGs, the shading cast by the PV panels inside would have the same effect on the crops, while part of the shaded solar radiation can be used to produce electricity.

The dynamic path of shading inside PVGs determines a decrease and a heterogeneous distribution of the solar radiation as a function of the greenhouse orientation, height, and percentage ratio of the area covered by PV panels projected on the greenhouse area, also called PV cover ratio (*PV*_*R*_) (Castellano, 2014; Cossu et al., 2018). Some design criteria have been identified to increase the agricultural sustainability of next-generation PVGs, such as keeping the *PV*_*R*_ under 20%, the application of semi-transparent PV technologies, the homogenous spatial distribution of the PV panels on the whole roof area (e.g., using a checkerboard pattern) and the taller greenhouse height (Minuto et al., 2009; Yano et al., 2010; Kadowaki et al., 2012; Ureña-Sánchez et al., 2012; Castellano, 2014; Blando et al., 2018; Yano and Cossu, 2019). Also the greenhouse North (N)-South (S) orientation can increase the availability of solar radiation for the crops, at a cost of a lower energy production (Cossu et al., 2018). These solutions can be applied only to new structures, while existing PVGs should be managed with different strategies, since modifying them would be economically prohibitive. Indeed, most existing structures were built with an excessive *PV*_*R*_ (equal or higher than 50%) to maximize the energy production and speculate on the incentives on PV energy, causing a considerable penalization of the crop productivity and a consequent agronomic underutilization of the PVG due to the excessive shading. This aspect causes an economic and environmental competition between the land use for energy toward food production that negatively affects the agronomic sustainability of the PVG.

To increase the agricultural productivity of the existing structures, it is important for the grower to investigate which greenhouse crops are suitable for cultivation, estimate the expected yield, and identify rotation plans depending on the available solar radiation. The suitable crops can be identified according to their light requirements and the solar radiation availability and distribution (Cossu et al., 2020). Plants can be classified into shade-tolerant and shade-avoidant species (Gommers et al., 2013). Shade-tolerant crops are capable of increasing the radiation interception efficiency (RIE) and radiation use efficiency (RUE) under shade, while shade-avoidant species react by concentrating the resources on stems and leaves, reducing the yield (Smith and Whitelam, 1997; Kläring and Krumbein, 2013). The persistent shading of the PV panels penalizes the productivity of horticultural species. A *PV*_*R*_ under 20% usually does not affect the yield of tomato, pepper, lettuce, zucchini, basil, and Welsh onion, even if the yield quality may decrease in terms of fruit size, color, and firmness, without affecting the marketable fraction (Minuto et al., 2011; Kadowaki et al., 2012; Ureña-Sánchez et al., 2012; Hassanien and Ming, 2017; Trypanagnostopoulos et al., 2017; Kavga et al., 2018; Aroca-Delgado et al., 2019). When the *PV*_*R*_ increases, a yield reduction proportional to the available light was reported on shaded tomato and lettuce inside PVGs or agrivoltaic systems (Marrou et al., 2013; Cossu et al., 2014; Bulgari et al., 2015). In addition, leafy vegetables under PV panels (lettuce and rocket) showed an increase in nitrate content due to a disproportion of nitrate ion uptake and metabolization that causes an accumulation in the leaves (Santamaria, 2006; Khan et al., 2018; Sirigu et al., 2019).

The common and green bean (*Phaseolus vulgaris* L.) are the main legume crops cultivated in Italy, with a production of 1.38 Mq in 2019 [ISTAT, (National agency for statistics), 2019]. Green bean is spread also in other southern EU countries such as Spain, where it is cultivated in open field (summer) and greenhouse (spring and autumn) and considered a high thermal and light demanding crop (López et al., 2008; Tesi, 2008). The effect of shading on common bean is characterized by a decrease in the yield and plant biomass proportional to the reduction of solar light, a decrease in the mean grain and shoot weight, coupled to an increase of the RUE and the specific leaf area (SLA), resulting in bigger and thinner leaves (Stirling et al., 1990; Tsubo and Walker, 2004; Hadi et al., 2006). The same effects were described on bean intercropped with maize in open field (Tsubo et al., 2001). A regressive crop model using the Gompertz function was proposed for the greenhouse green bean, based on the cumulated thermal time (López et al., 2008). However, by conducting a preliminary analysis, we found that this model did not result in reliable yield outputs in the range of the cumulated solar radiation inside PVGs, since the regression was calibrated on a conventional greenhouse.

At present, no data on green bean grown inside PVGs is available in literature. The aim of the present work is to fill this gap and determine the effect of PV shading on the yield, biomass partitioning and physiological adjustments of green bean inside a PVG with 50% *PV*_*R*_ located in Italy, also measuring the photosynthetic and transpiration rate. The experimental data were used to estimate the yield variation as a function of the available solar radiation and the agronomic sustainability toward the cultivation of green bean inside other common PVG types with *PV*_*R*_ ranging from 25 to 100%. The transpiration rate was used as basic data to discuss the potential application of precision irrigation technologies to PVGs, in order to optimize the water and nutrient use efficiency.

## Materials and Methods

### Photovoltaic Greenhouse and Experimental Crop

**FIGURE 1:**
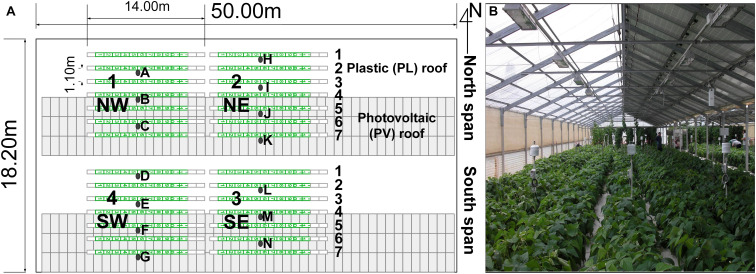
Photovoltaic greenhouse map **(A)**, plants rows (green lines) and experimental areas with sensor stations (black dots) and dwarf green bean crop inside the greenhouse **(B)**. Station D [under the plastic (PL) roof] and N [under the photovoltaic (PV) roof] were provided with additional photoradiometers for photosynthetic active radiation (PAR). Other experimental trials were performed in the background of the picture.

The green bean trials were conducted inside a gable roof PVG with two spans located in Decimomannu, southern Sardinia, Italy (39°19′59″N, 8°59′19″E). The greenhouse dimensions were 50.0 m × 19.2 m, area of 960 m^2^, 2.5-m gutter height, and a roof slope of 22° (Figure 1).

The greenhouse was East (E)-West (W) oriented, with North (N)-oriented roofs and sidewalls made with PVC (OndexBio, Renolit, France). The greenhouse was provided with a PV system formed by 288 multi-silicon opaque PV panels (REC 235PE, REC Solar, United States) that covered completely the South (S) oriented roofs, for a total power of 68 kWp and a *PV*_*R*_ of 50% (475 m^2^ of PV panels). The module efficiency (η_*m*_) was 14.2% and the energy loss due to the other components of the PV system (Balance of System, BOS) was estimated 15%. The overall efficiency of the PV system (η_*PV*_) can be estimated as the product of η_*m*_ and the efficiency of the BOS (85%), equal to 12.07%.

Dwarf green bean (*Phaseolus vulgaris* L., cv. Valentino) was used for all trials. Three crop cycles were conducted: cycle 1 in spring, from March 21 to June 3, 2013 (75 days); cycle 2 in summer from June 12 to August 5, 2013 (54 days); cycle 3 summer–autumn, from August 22 to October 25, 2013 (64 days). The plants were directly sown in bags (0.3 m × 1.0 m) filled with coconut fiber substrate (Coir EnGreen, Sri Lanka). In order to investigate the plant response in relation to the row position with respect to the PV roof, the crop was divided into four experimental areas (1–2 in the N span, 3–4 in the S span). Each experimental area was 11.0 m × 6.9 m (area 76 m^2^) and had seven plant rows: rows 1–3 under the plastic (PL) roof, row 4 under the ridge, rows 5–7 under the PV roof (Figure 1). The total crop area was 304 m^2^, and the plant density was 9.1 plants m^–2^. Since the row position with respect to the PV roof was the factor investigated, no randomization was adopted. Each plant row deployed 11 coconut fiber bags (Coir Engreen, Sri Lanka) and a row spacing of 1.1 m, for a total of 308 bags. Each coconut bag had 10 plants placed on a double row at 0.2-m distance. Additional coconut bags and plants were placed at the beginning and the end of the rows (total length of the row of 14.00 m) to replace plants with pathogens or explanted for the destructive measurements. A drip fertigation system supplied the following macronutrients and micronutrients (meq L^–1^): 12.3 N, 1.3 H_2_PO_4_^–^, 5.2 K^+^, 6.2 Ca^2+^, 4.6 Mg^2+^, 2.3 SO_4_^2–^, 0.032 Fe, 0.0012 Cu, 0.02 B, 0.011 Mn, and 0.005 Zn. A total irrigation volume of 775, 1,247, and 637 L m^–2^ was supplied, respectively, in cycles 1, 2, and 3. Side vents were located at the N and South (S) walls and regulated automatically with a set temperature of 25°C and no heating was supplied. The pest control was conducted with insect-proof nets on the side vents and applications of azadirachtin and abamectin, while fungal infections were prevented by one treatment with propamocarb at the planting date and foliar applications of copper and sulfur during the growing phase.

### Microclimate Monitoring

The microclimate conditions were measured using 14 custom-assembled sensor stations 1.0 m tall and placed on the N–S direction according to Figure 1 (one per row in each span), to measure the microclimate differences between plant rows. This positioning implied that the global irradiance (*I*_*g*_) was measured only on 14 of 28 plant rows to reduce the number of sensors. Indeed, previous studies conducted inside the same PVG demonstrated that the *I*_*g*_ was distributed on the transversal (N–S) direction, whereas the variability on the greenhouse length (E–W direction) was low (Cossu et al., 2017). Therefore, the *I*_*g*_ was assumed constant among the same rows of each span. Each sensor station was provided with a shielded thermohygrometer (Mela KPC2-ME, Galtec, Germany) and a silicon pyranometer for *I*_*g*_ (S-LIB-M003 Onset, Bourne, United States) connected to the same datalogger (Hobo microstation, Onset, Bourne, United States). In order to measure the average fraction of the photosynthetic active radiation (PAR) to the *I*_*g*_ (*f*_*p*_), the sensor stations D and N (Figure 1), located under the PL and PV roof, respectively, were provided with an additional photoradiometer for PAR radiation (S-LIA-M003 Onset, Bourne, United States). The fraction of PAR radiation inside the experimental PVG (*f*_*P*_) was determined for the rows under the PL (*f*_*PL*_, rows 1, 2, and 3) and the PV roofs (*f*_*PV*_, rows 4, 5, 6, and 7), by dividing the average PAR and *I*_*g*_ of the corresponding sensor station (D or N). Then, the corresponding fraction was multiplied for the average *I*_*g*_ data on an hourly basis of each pyranometer and on the *n* days of the cycle to calculate the cumulated PAR (*P*_*i*_) for all sensor stations, according to the following formula:

(1) $$P_{i}\;=\;\sum_{n\;=\;1}^{n}0.0036I_{g}(f_{PL}\;or\;f_{PV})\;{\text{(MJ m}}^{\text{-}}{}^{\text{2}}\text{)}$$

where the factor 0.0036 converts from W m^–2^ to MJ m^–2^. The external PAR radiation was calculated by multiplying the external *I*_*g*_ for the average PAR fraction for the sunlight of 0.48 (Amthor, 2010). An algorithm for the calculation of the solar radiation distribution inside PVGs (Cossu et al., 2017), already validated in the same experimental greenhouse, was used to elaborate a general function applicable at different latitudes that calculates the average percentage fraction of the greenhouse area shaded by the PV panels (*f*_*sh*_) as a function of the sun elevation angle (δ) at midday:

(2) $$F_{sh}\;=\;-0.00004{\text{δ}}^{2}-0.0028\text{δ}+0.8273\;(R^{2}\;=\;0.87)(\%)$$

This function is valid only for a gable roof greenhouse with a *PV*_*R*_ of 50%. According to this, the δ at midday ranged from 48.0° to 69.4° on cycle 1, from 63.3 to 69.9 on cycle 2, and from 36.3° to 58.7° on cycle 3. The external climate conditions were measured with a weather station 15 m far from the greenhouse, provided with a thermohygrometer (HOBOU10-003 Onset, Bourne, United States) and a pyranometer (LP Pyra 03, Delta Ohm, Padua, Italy). All parameters were measured at 15 min interval and averaged on hourly basis. The average greenhouse transmissivity (τ) was calculated from 7:00 to 20:00 using the *I*_*g*_ data on rows 1 (the least affected by the shading of the PV panels).

### Crop Monitoring

In each crop cycle, the total fresh and marketable yield was measured on 60 plants per row in each experimental area. The marketable yield was determined by the shape, size, and color features, excluding pods outside the commercial weight, rot or partially empty. At each harvest, the yield quality was assessed by measuring the average length and weight of 10 pods and the pod caliber on 10% of the marketable production. The pod caliber was classified according to two classes (<8.0 mm and ≥8.0 mm).

Destructive measurements were performed on cycles 1 and 3 (data not collected on cycle 2) at 2-week intervals on two plants of rows 2 (under the PL roof), rows 4 (at the center of the span under the ridge), and rows 6 (under the PV roof). The average fresh (*W*_*f*_) weight of the plant organs (leaves, stems, and pods), number of leaves, pods, and leaf area (*A*) were measured. The dry biomass was determined after drying the samples in oven at 70°C till constant weight. The leaf area was measured using an optical planimeter (Li-3100 C, Li-cor, Lincoln, United Kingdom). In addition, the following leaf parameters were calculated: leaf area index (LAI), net assimilation rate (NAR), specific leaf area, (SLA) and leaf area ratio (LAR). NAR is the net plant mass increase per unit of leaf area, and it is the result of biomass gain between photosynthesis and respiration (Li et al., 2016). The NAR between two sampling dates was calculated with the following formula:

(3) $$NAR\;=\;\frac{W_{d2}-W_{d1}}{A_{2}-A_{1}}{\text{(g m}}^{\text{-}}{}^{\text{2}}\text{)}$$

where the difference of plant *W*_*d*_ and *A* between sampling dates was expressed with the subscript 2 and 1. The first NAR data (day 19) was calculated assuming an average LAI of 0.10 and an average *W*_*d*_ of 0.15 g at day 1. SLA is the ratio of *A* and the leaf dry weight (*W*_*l*_) and can be considered a measure of the leaf thickness (Vile et al., 2005):

(4) $$SLA\;=\;\frac{A}{W_{l}}{\text{(cm}}^{\text{2}}{\text{ g}}^{\text{-}}{}^{\text{1}}\text{)}$$

LAR is the ratio of the total leaf area and the plant dry mass (Allaby, 2006):

(5) $$LAR\;=\;\frac{A}{W_{d}}{\text{(cm}}^{\text{2}}{\text{ g}}^{\text{-}}{}^{\text{1}}\text{)}$$

The radiation use efficiency (RUE) can be calculated as the slope of the linear regression between the total fresh or dry yield and the cumulated PAR (Muchow et al., 1993; Muchow and Sinclair, 1994; Tei et al., 1996; Tesfaye et al., 2006). According to this, the cumulated PAR (*P*_*i*_) was used to calculate the RUE on fresh yield (RUE_*F*_) with the following ratio:

(6) $$RUE_{F}\;=\;\frac{Y_{t}}{\sum_{n=1}^{n}P_{i}}{\text{(g MJ}}^{\text{-}}{}^{\text{1}}\text{)}$$

where *Y*_*t*_ is the total fresh yield, and *P*_*i*_ is cumulated on the *n* days of the cycle.

The PAR radiation at leaf surface, the photosynthetic net assimilation and transpiration rate were measured on one plant per row in each experimental area, between 10:00 and 14:00, on 4 days of cycle 1 (April 29, May 7, May 27, and June 4, 2013) and 3 days of cycle 3 (September 26, October 10, and October 24, 2013), using a portable infrared gas analyzer for photosynthesis (CIRAS-2, PP Systems, Amesbury, United States).

### Estimation of the Green Bean Yield Inside the Photovoltaic Greenhouse Types

**FIGURE 2:**
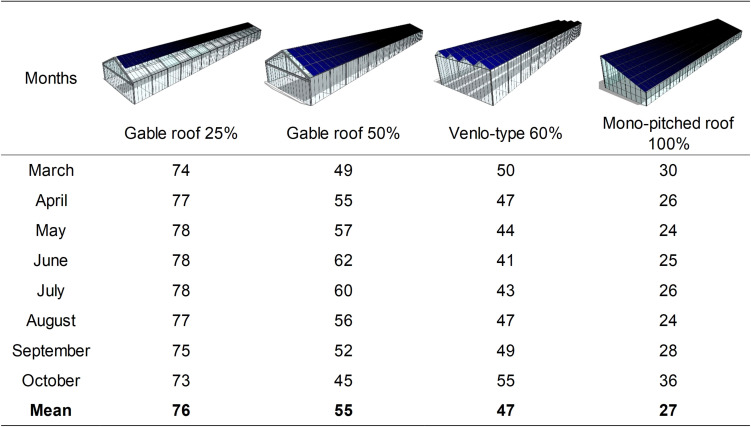
Average monthly percentage of available cumulated PAR from March to October compared with a conventional greenhouse without PV panels on the roof, expressed as GGR coefficients for four photovoltaic greenhouse (PVG) types with E–W orientation. The coefficients were calculated at 1.5 m above ground level and retrieved from the literature (Cossu et al., 2018).

The yield response of green bean crop inside the experimental PVG was determined with a multiple linear regression model based on all cycles with three variables: the *P*_*i*_, the daily temperature sum (*T*_*s*_) and the average relative humidity (*RH*_*a*_) of each plant row:

(7) $$Y_{t}\;=\;f\left(P_{i1},P_{i2}\ldots T_{s1},T_{s2}\ldots RH_{a1},RH_{a2}\ldots \right)\;=\;aP_{i}+bT_{s}+cRH_{a}+d{\text{(kg m}}^{\text{-}}{}^{\text{2}}\text{)}$$

where *a*, *b*, and *c* are the related regression coefficients for *P_i_*, *T_s_*, and *RH_a_*, respectively. The microclimate data of each sensor station was used for the two corresponding rows of the same span, since only one station per row was available. *T_s_* was calculated using the Monteith expression (Monteith et al., 1977):

(8) $$T_{s}\;=\;\sum_{n\;=\;1}^{n}(T_{m}-T_{b}){\text{(}}^{\text{°}}\text{C days)}$$

where *T*_*m*_ is the mean daily temperature, and *T*_*b*_ is the base temperature assumed 4.2°C for green bean (Ferreira, 1997). The linear regression of Eq. 7 was used to estimate the yield inside the other three PVG types available in literature: a gable roof greenhouse with 25% PV cover ratio with the same structure of the PVG considered in this study, a venlo-type greenhouse with 60% *PV*_*R*,_ and a single pitched-roof greenhouse with 100% *PV*_*R*_, as depicted in Figure 2.

These PVG types are spread in southern European countries and their solar radiation distribution data are already available in literature (Cossu et al., 2018). Each type had a gutter height of 2.5 m (except the type with *PV*_*R*_ of 60%, with a height of 4.5 m) and were characterized by specific percentage ratios of available PAR radiation compared with a conventional greenhouse without PV panels on the roof (*G*_*GR*_), calculated on a monthly basis. To estimate the yield in the other PVG types, their available *P*_*i*_ was calculated for each cycle starting from the daily external cumulated PAR (*P*_*o*_):

(9) $$P_{i}\;=\;\sum_{n\;=\;1}^{n}\left(P_{o}\cdot 0.0036\cdot \tau \cdot G_{GR}\right)\text{ (MJ }{\text{m}}^{\text{-}}{}^{\text{2}}\text{)}$$

where 0.0036 is a coefficient from Wh m^–2^ to MJ m^–2^, the average τ was determined experimentally and assumed constant, whereas the *G*_*GR*_ changed on a monthly basis according to the coefficients reported in Figure 2.

The specific economic revenue of the yield per square meter (calculated from the *RUE*_*F*_) was compared with the value of the PV energy produced by the PVG per square meter, considering an average price of the green bean in Italy of 1.5 € kg^–2^ (ISMEA, Istituto di Servizi per il Mercato Agricolo Alimentare, 2021) and an average price of the electricity of 0.622 € kWh^–1^, which is the sum of the feed-in tariff of the incentive granted for 20 years to the farm (0.422 € kWh^–1^) and the average price of the electricity in Italy (0.20 € kWh^–1^) (ARERA, 2021), which can be intended also as an energy saving, since the greenhouse self-consumed its PV energy. The electricity produced by the PV system was retrieved from the inverter.

### Statistical Analysis

Statistical analysis of the crop data was carried out using one-way ANOVA with the row position as treatment (seven rows for yield and physiological variables and three rows for plant growth parameters) and four replicates (experimental areas). The LSD test determined the statistical differences between rows at *P* < 0.05 significance level. To highlight the spatial variability of the *P*_*i*_ and the *Y*_*i*_ between rows the coefficient of variation (CV) was calculated as the ratio of the standard deviation and the mean of the rows. The statistical analysis was conducted using the Minitab statistical software (Minitab 17 Statistical Software, 2010. State College, United States. Minitab, Inc.).

## Results

### Photovoltaic Greenhouses Microclimate

**FIGURE 3:**
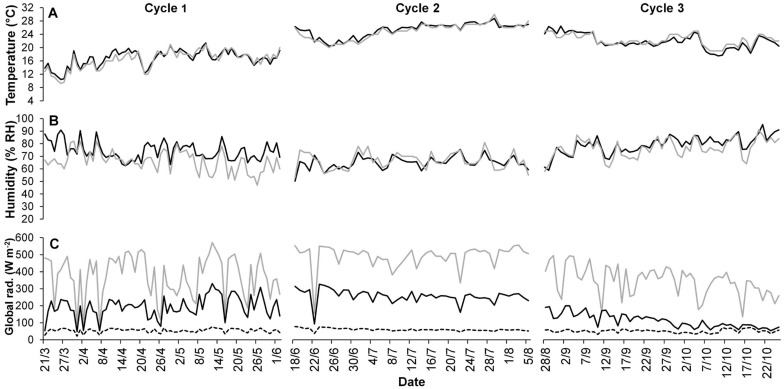
Average daily temperature **(A)** and humidity **(B)** inside the greenhouse (black line) and outside (gray line) during the three crop cycles. The average daily global irradiance (*I*_*g*_) **(C)** is depicted under the plastic cover (black line), the photovoltaic cover (dotted line) and outside (gray line).

The average microclimate conditions inside the PVG are summarized in Figure 3.

The average daily greenhouse temperatures were 16.5°C, 25.1°C, and 22.3°C, respectively, for cycles 1, 2, and 3, corresponding to an average *T*_*s*_ of 1,049°C, 1,201°C, and 1,107°C days, respectively. The *RH*_*a*_ was 75.1, 64.4, and 75.0% for cycles 1, 2, and 3, respectively. The average greenhouse temperature was 0.4–0.6°C higher than outside on cycles 1 and 2, up to 3.5°C during specific days of cycle 3. The average external *I*_*g*_ ranged from 345 W m^–2^ of cycle 3 to 490 W m^–2^ of cycle 1, whereas it was, respectively, 78 and 150 W m^–2^ inside the greenhouse (Figure 3C). The *I*_*g*_ under the PL roof was 69% higher than the PV roof on average. Both the τ and *F*_*sh*_ were 55%, as the average of all cycles. The *F*_*sh*_ ranged from 48.3% on the summer cycle (cycle 2) to 60.7% on the autumn cycle (cycle 3). The average *f*_*P*_ was 0.45, as the average of all cycles, ranging from an *f*_*PV*_ of 0.43 to an *f*_*PL*_ of 0.46. The average *P*_*i*_ was 69% lower than the external cumulated PAR and 45% lower than a hypothetic conventional greenhouse with the same τ of 55% (Table 1).

**TABLE 1:** Average yield data per row after the three cycles.

	Plant rows							Mean	CV
	1	2	3	4	5	6	7		
*Cycle 1 (75 days)*									
Cumulated PAR (MJ m^–2^)	404	371	144	123	80	83	84	184	77%
Total yield (kg m^–2^)	2.50^a^	2.46^a^	1.55^b^	1.11^c^	0.98^cd^	0.81^d^	1.08^cd^	1.50	47%
Marketable yield (kg m^–2^)	2.36^a^	2.32^a^	1.48^b^	1.04^c^	0.91^c^	0.76^c^	1.02^c^	1.41	47%
Pod length (cm)	13.7^a^	13.7^a^	13.6^b^	13.1^c^	13.0^c^	13.1^c^	13.2^bc^	13.4	2%
Pod fresh weight (g)	6.4^a^	6.1^a^	5.4^b^	4.8^c^	4.8^c^	4.8^c^	5.2^bc^	5.4	12%
*Caliber class (%)*									
<8.0 mm	35^a^	36^a^	43^ab^	52^bc^	56^c^	57^c^	52^bc^	47	19%
>8.0 mm	65^a^	64^a^	57^ab^	48^bc^	44^c^	43^c^	48^bc^	53	17%
*Cycle 2 (54 days)*									
Cumulated PAR (MJ m^–2^)	463	405	144	117	80	78	72	208	85%
Total yield (kg m^–2^)	2.74^a^	2.49^a^	2.00^b^	1.79^b^	1.08^c^	0.79^d^	1.13^c^	1.72	43%
Marketable yield (kg m^–2^)	2.57^a^	2.39^a^	1.71^b^	1.63^b^	0.94^c^	0.71^d^	1.01^c^	1.61	43%
Pod length (cm)	13.4^a^	13.3^a^	13.5^a^	13.4^a^	13.1^b^	12.8^b^	12.8^b^	13.2	2%
Pod fresh weight (g)	5.7^a^	5.6^a^	5.1^a^	4.9^a^	4.6^b^	4.5^b^	4.7^b^	5.0	9%
*Caliber class (%)*									
<8.0 mm	12^a^	38^a^	53^b^	57^bc^	61^bc^	65^c^	57^bc^	52	24%
>8.0 mm	69^a^	62^ab^	47^*abc*^	43^bc^	39^bc^	35^c^	43^bc^	48	26%
*Cycle 3 (64 days)*									
Cumulated PAR (MJ m^–2^)	253	144	75	72	53	69	248	131	66%
Total yield (kg m^–2^)	1.24^a^	0.86^b^	0.73^b^	0.61^b^	0.56^b^	0.66^b^	1.42^a^	0.87	38%
Marketable yield (kg m^–2^)	1.14^a^	0.78^b^	0.67^b^	0.55^b^	0.52^b^	0.61^b^	1.32^a^	0.80	39%
Pod length (cm)	13.5^a^	13.1^b^	13.3^b^	12.8^b^	12.7^b^	13.2^b^	13.5^a^	13.1	3%
Pod fresh weight (g)	4.7^b^	4.3^b^	4.3^b^	4.3^b^	4.2^b^	4.6^b^	5.6^a^	4.6	11%
*Caliber class (%)*									
<8.0 mm	63^a^	68^a^	68^a^	72^a^	75^a^	63^a^	45^b^	65	15%
>8.0 mm	37^a^	32^a^	32^a^	28^a^	25^a^	37^a^	55^b^	35	28%

*Each plant row is the average of the four experimental areas (four replicates), except for the cumulated photosynthetic active radiation (PAR) (two replicates). Mean and coefficient of variation (CV) of all the rows are also included. Means that do not share a letter are statistically different (LSD test *p* < 0.05).*

The *P*_*i*_ was heterogeneously distributed on the greenhouse area with an average CV of 76% between rows, as the average of all cycles. The *P*_*i*_ resulted higher under the PL roof, with maximum values on rows 1 and decreased gradually throughout the span width (N-S direction) till the minimum values of rows 5 or 6. The *P*_*i*_ under the PV roof (rows from 5 to 7) was 75% lower than the rows under the PL roof (rows from 1 to 3), as the average of cycle 1 and 2. On the other hand, during cycle 3 the *P*_*i*_ showed a more homogenous distribution (CV of 66%) and it was only 22% higher under the PL roof than the PV roof.

### Green Bean Yield Distribution and Radiation Use Efficiency

The average *Y*_*t*_ was 1.36 kg m^–2^, distributed as 1.50, 1.72, and 0.87 kg m^–2^ for cycles 1, 2, and 3, respectively (Table 1). No statistical differences were found between experimental areas. The *Y*_*t*_ followed the same distribution of the *P*_*i*_, resulting higher under the PL roof (maximum yield observed on rows 1) and decreasing proportionally on the rows under the PV roof. On average, the *Y*_*t*_ under the PV roof (rows from 5 to 7) was 49% lower than the PL roof (rows from 1 to 3). The highest yield was observed on the rows farthest from the PV cover and decreased gradually throughout the span (N–S direction). An exception was observed on cycle 3, where the most productive rows were 1 and 7, statistically different from the other rows. The average CV of the yield between rows was 43%, with the lowest value observed on cycle 3 (38%). The marketable production was averagely 93%.

The average pod caliber was distributed almost equally among the two classes, except on cycle 3, in which they resulted significantly smaller, with 65% of the pods in the lower class (<8.0 mm). The pod caliber was generally higher under the PL roof than the PV roof, with the prevalence of the higher caliber class (≥8.0 mm), especially on cycle 2.

The fresh yield followed a multiple linear regression as a function of the monitored microclimate parameters *P*_*i*_, *T*_*s*,_ and *RH*_*a*_:

(10) $$Y_{f}\;=\;4.96-0.004418P_{i}-0.04131RH_{a}-0.001266T_{s}\;(R^{2}\;=\;0.83){\text{(kg m}}^{\text{-}}{}^{\text{2}}\text{)}$$

The mean absolute error (MAE) was 0.04 kg m^–2^, and the root mean square error (RMSE) was 0.25 kg m^–2^, equal to 18% of the average yield of the three cycles (1.36 kg m^–2^). The yield variation as a function of 1% *P*_*i*_ variation was equal to the *P*_*i*_ coefficient of Eq. 10 (0.44%) as the average of all cycles (assuming constant *T*_*s*_ and *RH*_*a*_). Eq. 10 was used to calculate the yield reduction inside the other PVG types with *PV*_*R*_ ranging from 25% to 100% (Table 2).

**TABLE 2:** Expected average green bean yield in the four photovoltaic greenhouse (PVG) types with photovoltaic cover ratio (*PV*_*R*_) ranging from 25 to 100%.

Cycles	Conventional greenhouse	Gable roof 25%	Gable roof 50%	Venlo-type 60%	Mono-pitched 100%
*Cycle 1 (T_*s*_ = 1,049; RH_*a*_ = 75.1%)*					
Cumulated PAR *P*_*i*_ (MJ m^–2^)	324	251	184	146	81
Expected yield *Y*_*t*_ (kg m^–2^)	1.96	1.64 (−16%)	1.50 (−23%)	1.17 (−40%)	0.89 (−55%)
*Cycle 2 (T_*s*_ = 1,201; RH_*a*_ = 64.4%)*					
Cumulated PAR *P*_*i*_ (MJ m^–2^)	364	284	208	155	93
Expected yield *Y*_*t*_ (kg m^–2^)	2.39	2.03 (−15%)	1.72 (−28%)	1.46 (−39%)	1.19 (−50%)
*Cycle 3 (T_*s*_ = 1,107; RH_*a*_ = 75.0%)*					
Cumulated PAR *P*_*i*_ (MJ m^–2^)	256	191	131	129	76
Expected yield *Y*_*t*_ (kg m^–2^)	1.59	1.30 (−18%)	0.87 (−45%)	0.86 (−46%)	0.80 (−50%)
*Average values*					
PAR reduction (%)	−	23	45	54	73
Expected yield (kg m^–2^)	1.98	1.66	1.36	1.17	0.96
Yield reduction (%)	−	16	31	41	52
*Estimated total income (€ m^–2^)*					
Green bean	8.91	7.46	6.14	5.25	4.31
PV energy	−	20.53	41.05	49.26	82.10
Total	8.91	27.99	47.19	54.51	86.41

*The gable roof PVG with 50% *PV*_*R*_ reports the experimental cumulative PAR inside the PVG (*P*_*i*_) and yield data of Table 1 for comparison. The total fresh yield (*Y*_*t*_) is calculated using the reported multiple linear regression of Eq. 7 and compared with the hypothetic conventional greenhouse (τ = 55%) reporting the percentage reduction in brackets. The yield estimations in these types assumed the same average daily mean temperature (*T*_*m*_) and *RH*_*m*_ and humidity occurred of the respective crop cycles indicated in brackets. The photovoltaic (PV) energy income was 0.622 € kWh^–1^ (price + feed-in tariff) and the average price of green bean 1.5 € kg^–1^.*

The *Y*_*t*_ measured inside the PVG with a *PV*_*R*_ of 50% suffered from an average yield reduction of 31% compared with the conventional greenhouse (estimated 1.96 kg m^–2^ on average). By applying Eq. 10, this average reduction was estimated up to 52% for the PVG type with *PV*_*R*_ of 100%. The lowest green bean performance was estimated in the autumn (cycle 1) inside the PVG type with *PV*_*R*_ of 100%, with 55% yield reduction. Only the PVG type with a *PV*_*R*_ of 25% achieved a satisfactory yield with a limited average yield reduction of 16%. In cycle 3, the PVG type with *PV*_*R*_ of 60% showed a *P*_*i*_ and *Y*_*t*_ comparable with the PVG with *PV*_*R*_ of 50% (0.87 and 0.86 kg m^–2^, respectively), equal to 46% yield reduction. Cycle 3 achieved the worst green bean performance due to the high *F*_*sh*_ and the lowest *P*_*i*_.

The total income of the PVG (crop and PV energy) in the three cycles was 47.19 € m^–2^, which was 429% higher than that of the conventional greenhouse (only crop), equal to 8.91 € m^–2^ (Table 2). There was an inverse relation between the income from PV energy and crop as a function of the *PV*_*R*_. The highest income was reached by the PVG type with *PV*_*R*_ of 100%, since the higher the *PV*_*R*_, the higher the income from PV energy, which abundantly compensated the income reduction of the crop. Based on the table data, the two incomes (green bean and PV energy) became equal when the *PV*_*R*_ was 10%.

**FIGURE 4:**
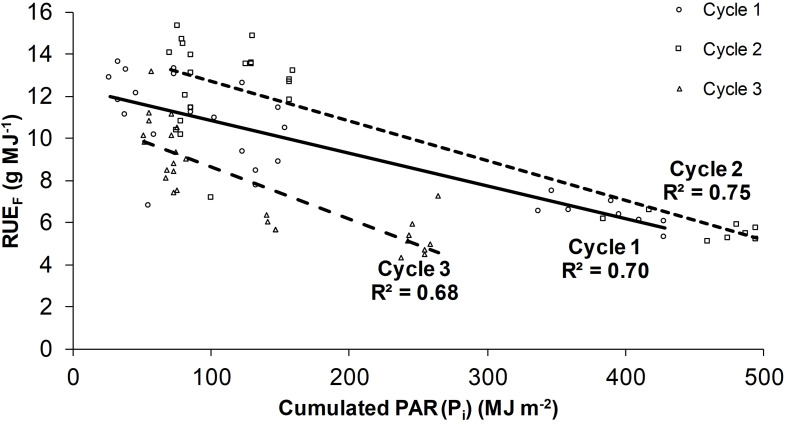
Radiation use efficiency on fresh weight (*RUE*_*F*_) as a function of the cumulated PAR radiation (*P*_*i*_). The linear regressions are the following: Cycle 1 (*RUE*_*F*_ = −0.0156 *P*_*i*_ + 12.144); Cycle 2 (*RUE*_*F*_ = −0.0189 *P*_*i*_ + 14.619); Cycle 3 (*RUE*_*F*_ = −0.0246 *P*_*i*_ + 11.094). The regressions of each cycle are specific for the values of daily temperature sum (*T*_*s*_) and average relative humidity (*RH*_*a*_) indicated in paragraph 3.1.

The average *RUE*_*F*_ was 9.4 g MJ^–1^, in particular 9.7, 10.7, and 7.9 g MJ^–1^ on cycles 1, 2, and 3, respectively, according to the microclimate conditions. An inverse relation between *RUE*_*F*_ and *P*_*i*_ was observed, showing a higher efficiency in the plant rows with a lower *P*_*i*_ (rows under the PV roof) (Figure 4). The *RUE*_*F*_ difference between the rows under the PL and PV roof ranged from 7% to 61% depending on the season (higher in summer and lower in autumn).

Rows 1 and 2 had the lowest values (6.4 g MJ^–1^ as the average of the three cycles), whereas it was 66% higher on the rows under the PV roof, especially rows 5 and 7 (10.6 g MJ^–1^ on average). To compare the efficiency of the crop and the PV system in economic terms, the average *RUE*_*F*_ of the three cycles (9.4 g MJ^–1^) was multiplied for the cumulated *P*_*i*_ and divided to the total electricity production (66 kWh m^–2^), resulting in 74.5 g kWh^–1^. This productivity was equal to 11.2 € kWh^–1^, thus 82% lower than the income of the PV energy (feed-in tariff + electricity price of 62.2 € kWh^–1^). Compared with a conventional greenhouse, the cumulated average *Y*_*t*_ reduction of the three cycles (31%) was 1.85 kg m^–2^, which corresponded to 4.2 c€ m^–2^ of green bean income lost for each kWh of PV energy.

### Biomass Parameters and Distribution

**FIGURE 5:**
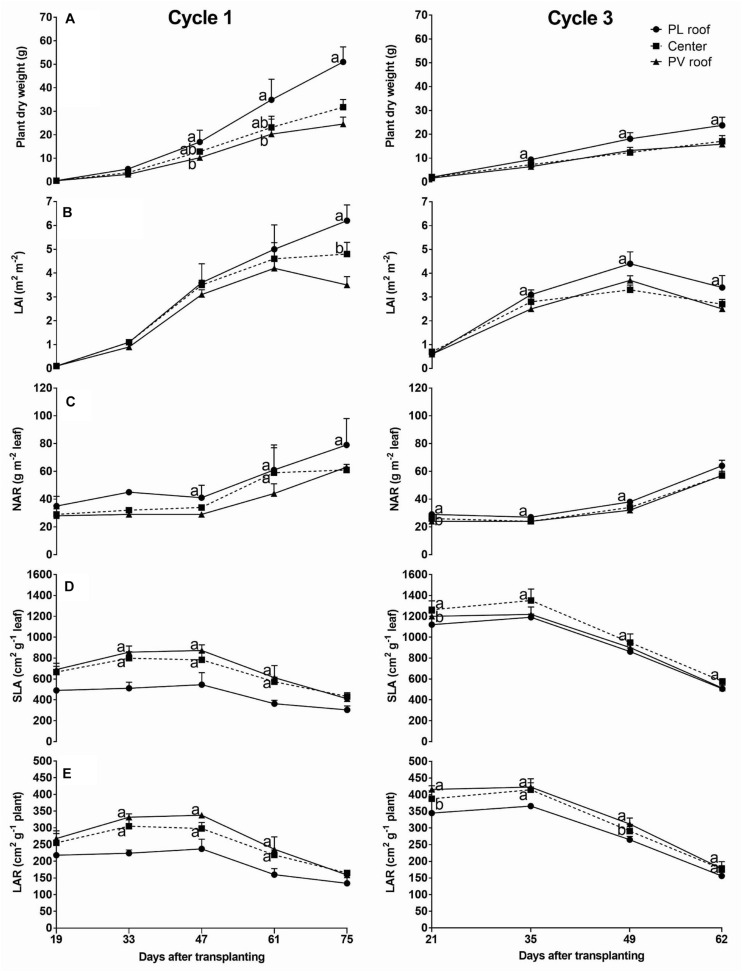
Plant dry weight and leaf parameters on 5 days of cycle 1 (from day 19 to 75) and 4 days on cycle 3 (from day 21 to 62). Parameters reported: plant dry weight **(A)**, leaf area index (LAI) **(B)**, net assimilation rate of the leaf (NAR) **(C)**, specific leaf area (SLA) **(D)**, and leaf area ratio (LAR) **(E)**. Each data is the average of eight plants (two for each experimental area) taken under the PL roof (rows 2, black circles), under the center of the greenhouse span (rows 4, black squares) and under the PV roof (rows 6, black triangles). The upper bars represent the standard deviation. Symbols in the same day that do not share a letter or a missing letter are significantly different (LSD test *p* < 0.05).

The plant dry weight accumulation followed the increase of *P*_*i*_ and was 52 and 33% lower under the PV roof (rows 6) than under the PL roof (rows 2), at the end of cycles 1 and 3, respectively (Figure 5A).

The plant dry weight and leaf parameters observed under the PV roof (sample plants on rows 6) and the center of the greenhouse span (rows 4) were usually not significantly different from each other. Both LAI and NAR increased till harvest and the values under PV roof resulted lower than the PL roof at the end of cycle 1 by 43 and 39%, respectively, followed by 24 and 12% at the end of cycle 2 (Figures 5B,C). On the contrary, the rows under the PV roof showed a SLA and LAR higher than the PL roof on both cycles (Figures 5D,E). In particular, the SLA difference between PV and PL roof occurred in the central part of the cycle, up to 32% on day 61 of cycle 1 and 15% on day 49 of cycle 3, whereas it was smaller at harvest. The LAR followed a similar trend, with values up to 32% higher under the PV roof than the PL roof on day 61 of cycle 1. The SLA and LAR were, respectively 39 and 24% higher in cycle 3 than cycle 1.

**FIGURE 6:**
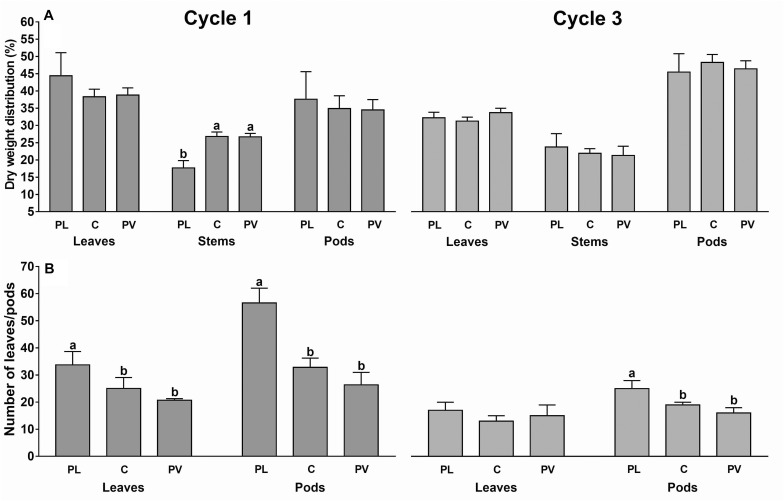
Percentage dry weight distribution in the plant organs **(A)** and average number of leaves and pods per plant **(B)** under the plastic roof (rows 2, PL), center of the greenhouse span (rows 4, C) and photovoltaic roof (rows 6, PV). Data are referred to cycle 1 and 3 (cycle 2 data not retrieved). Each bar is the average of eight sample plants (two for each experimental area). The upper bars represent the standard deviation. Bars that do not share a letter are significantly different (LSD test *p* < 0.05).

Cycle 3 showed a dry weight distribution on the pods 24% higher than cycle 1, while it was lower in leaves and stems (Figure 6A).

The dry matter was distributed in leaves (36.8%) and pods (40.9%) and only the remaining 22.2% was in the stems. In particular, only during cycle 1, the dry weight distribution in the stems was 34% higher under the PV roof than the PL roof. The number of leaves and pods were, respectively, 25 and 35% higher under the PL roof than the PV roof, as the average of both cycles (Figure 6B).

### Photosynthetic and Transpiration Rate

**FIGURE 7:**
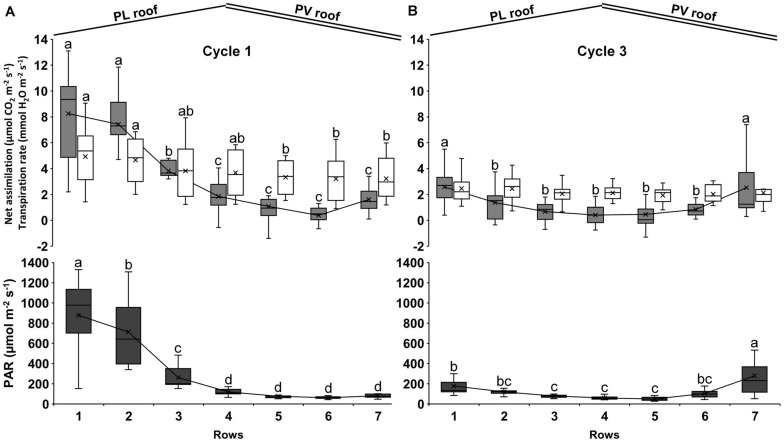
Average net photosynthetic assimilation rate (gray bars), transpiration rate (white bars) and PAR radiation (black bars) during cycle 1 **(A)** and cycle 3 **(B)**. The measurements were performed on one plant per row from 10:00 to 12:00 and comprise 4 days during cycle 1 (April 29, May 7, May 27, and June 4, 2013) and 3 days on cycle 3 (September 29, October 10, and October 24, 2013). Each bar represents the data distribution on the row: the upper and lower parts are the first and last quartile, respectively, the internal line is the median, and the cross is the mean. The upper and lower error bars are the maximum and minimum values, respectively. Each bar includes the four corresponding rows of each experimental area. Bars that do not share a letter are significantly different (LSD test *p* < 0.05).

The distribution of the net assimilation and transpiration rate is depicted in Figure 7.

Both parameters followed the gradient of heterogeneous distribution of the PAR between rows. During cycle 1, the average PAR under the PV roof (rows from 5 to 7) was 73 μmol m^–2^ s^–1^, thus 88% lower than the PL roof (rows from 1 to 3), where the average PAR was 618 μmol m^–2^ s^–1^ (Figure 7A). The rows under the PV roof showed an average net assimilation of 1.0 μmol CO_2_ m^–2^ s^–1^, which was 84% lower than under the PL roof (6.5 μmol CO_2_ m^–2^ s^–1^). Rows 1 attained the highest net assimilation with values up to 13.1 μmol CO_2_ m^–2^ s^–1^. In addition, rows 1 showed the highest variability between days, with considerable differences between the first and last quartile and a CV of 47%. Rows 5 and 6 showed the minimum net assimilation, with negative values up to −1.4 μmol CO_2_ m^–2^ s^–1^. The transpiration rate under the PV roof (3.3 mmol H_2_O m^–2^ s^–1^) was 27% lower than under the PL roof (4.5 mmol H_2_O m^–2^ s^–1^), with a CV of 48% on average. The maximum transpiration rate was recorded along row 1, with 9.1 mmol H_2_O m^–2^ s^–1^ and the minimum was observed under row 6, with 0.9 mmol H_2_O m^–2^ s^–1^.

With regard to cycle 3 measurements, the PAR was distributed mainly on the sidewalls of the span (on rows 1 and 7) (Figure 7B). The average PAR under the PV roof was 144 μmol m^–2^ s^–1^ in the 3 days of measurements and 15% higher than the PL roof (125 μmol m^–2^ s^–1^), with a CV of 33% as the average on all rows. Rows 7 showed the maximum value, with 279 μmol m^–2^ s^–1^ as the average of the 3 days of measurement. The average net assimilation under the PV roof (1.5 μmol CO_2_ m^–2^ s^–1^) was 15% lower than under the PL roof (1.8 μmol CO_2_ m^–2^ s^–1^), but the differences between rows were not significant from rows 2 to 6. Negative net assimilation rates were observed both under the PV and PL roof, including −0.4 μmol CO_2_ m^–2^ s^–1^ on rows 2 and up to −1.3 μmol CO_2_ m^–2^ s^–1^ on rows 5. The average transpiration rate under the PV roof was 2.3 mmol H_2_O m^–2^ s^–1^, thus 8% lower than what was observed along the rows under the PV roof, where it was 2.5 mmol H_2_O m^–2^ s^–1^ on average, but the difference between rows did not result statistically significant.

## Discussion

### Effects of the Microclimate and Cumulated Photosynthetic Active Radiation Distribution on Green Bean Yield

The PV panels contributed to reduce the greenhouse effect, with only 0.4–0.6°C difference between the average external and internal temperature (Figure 3). Both the average temperature range of the three cycles (from 16.5°C to 25.1°C) and the average *T*_*s*_ range (from 1,049°C to 1,201°C days) were lower than the optimal temperature (25°C) and *T*_*s*_ of 1,800–2,000°C days of green bean (Baudoin et al., 2017), indicating that temperature was not excessive for the crop. The cooling effect of the PV panels is well known, and it can be useful to manage and reduce the greenhouse thermal load during summer (Hammam et al., 2007; Chemisana, 2011). This benefit has the cost of shading the solar radiation even during periods of the day in which it does not saturate the plant photosystem (Stanghellini, 2011). Both temperature and humidity were homogeneously distributed on the greenhouse environment due to the automatic controlled side-vent openings, that were often open during summer and that contributed to decrease the temperature and humidity differences compared with outside. Only during cycle 3 the temperature was higher than outside in several days (with a maximum difference of 3.5°C), due to the more frequent closure of the side vents.

The average *I*_*g*_ was distributed mainly under the PL roof and 69% higher than the PV roof on average, due to the shading of the PV panels. During cycles 1 and 2 a remarkable difference of 75% was observed between the *P*_*i*_ under the PL and PV roof that decreased to 22% in cycle 3 (Figure 3C and Table 1). Indeed, former studies conducted inside the same E-W oriented PVG showed that the shadow of the panels follows a dynamic path during the year, casting mainly under the panels for most of the year, except during autumn and winter, where it casts also under the PL roof (Cossu et al., 2017). This difference was determined by δ, which reached low values in winter, causing the shadow to cast on a higher percentage of the greenhouse area (higher *F*_*sh*_) according to Eq. 2. In particular, *F*_*sh*_ reaches the highest value (73%) at δ = 26° at midday, which corresponded to December. When low δ values occur, the shadow covers more than 50% of the PVG area and casts also under the PL roof during winter and part of autumn. On the contrary, in summer the average δ is higher (maximum value of 70° at midday observed in June), leading to a lower *F*_*sh*_ around 44%. As a consequence, the seasonal effect on the PV shading determines a *I*_*g*_ on the greenhouse area more homogeneous in autumn and winter, reducing the variability between PL and PV roof (CV of 66% in cycle 3), compared with spring and summer (CV of 77 and 85% in cycle 1 and 2, respectively). This explained the different *Y*_*f*_ distribution between rows on cycle 3 compared with the other two cycles.

The average *Y*_*t*_ of the three cycles (1.36 kg m^–2^) was 31% lower than the green bean grown in a cooled greenhouse, where it was 2.00 kg m^–2^ per season (Riccardi, 2009; Castilla et al., 2012; Nejatian, 2017). This latter value is in agreement with the estimated value of 1.94 kg m^–2^ inside the conventional greenhouse (Table 2). On the other hand, the *Y*_*t*_ distribution during cycle 3 was different due to the dynamic path of the shading in autumn, with rows 7 showing the highest yield and a more uniform distribution between rows. Indeed, since the *I*_*g*_ was distributed differently depending on the season and it was more homogeneous during autumn, a reduction of the yield variability between the rows under the PL and PV roof was observed during cycle 3. Other studies on green bean in a greenhouse in Mediterranean climate reported an average *Y*_*t*_ of 2.71 kg m^–2^ (González et al., 2009; Baudoin et al., 2017), in line with what was observed on the rows under the PL roof during cycle 1 and 2, where it ranged from 2.46 to 2.74 kg m^–2^. As a consequence, the *Y*_*t*_ of the three rows under the PL roof were the least affected by the PV shading, with an average reduction ranging from 11 to 20%, respectively, in cycles 2 and 1, compared with a conventional greenhouse. This aspect highlights that in spring and summer the *P*_*i*_ under the PL roof allows to obtain a *Y*_*t*_ comparable with a conventional greenhouse, with none or limited yield reduction.

The *RUE*_*F*_ increased with the average *T*_*s*_ resulting higher in the summer cycle and lower in autumn, indicating that the temperature was not excessive and was a limiting factor affecting the efficiency of the plant between cycles. The *RUE*_*F*_ under the PV roof increased averagely by 36% compared with the rows under the PL roof, and ranged from 26 to 48% depending on the season, similarly to what was observed on common bean intercropped with maize in open field, which showed an increase of RUE up to 77% (Tsubo and Walker, 2004). Furthermore, plants increased the dry matter partitioning to stems and leaves by 50% compared with the non-shaded crop. It should be noted that the RUE of the common bean is also affected by the plant density, resulting 20% higher with a plant density of 40 plants m^–2^, compared with a lower value of 13 plants m^–2^ (Ghavidel et al., 2016). The RUE increased due to the higher SLA (Figure 5D). In fact, the plants under shading invest their photosynthetic products in the increase of leaf area at the cost of a thickness reduction and this was already observed on bean (Hadi et al., 2006; Lambers et al., 2008). Other previous trials on common bean showed an increase of both SLA and LAR as a bean acclimation feature due to the reduced irradiation (Worku et al., 2004). Recent experiments on winged bean under shading nets observed that the lower light intensity contributed to delay flowering and that moderately shaded plants with 30% shading nets achieved higher yield than non-shaded plants (Raai et al., 2020). However, the common shading applied using shading nets or during intercropping is considerably different from the shading of a PVG, which is higher and persistent, since opaque PV panels shade the direct radiation completely. For this reason, semi-transparent and organic PV technologies are recommended for PVG applications to reduce the impact on the available irradiation and add photo-selective properties that can enhance the crop growth (Li et al., 2018; Baxevanou et al., 2020; Friman-Peretz et al., 2020).

### Effect of the Photovoltaic Greenhouses Type, Orientation, and Height on Green Bean Yield

The considerable disproportion between the specific income from green bean and PV energy production (the latter four times higher inside the experimental PVG with a *PV*_*R*_ of 50%) is the main drawback of the PVGs, since it poses the problem of identifying suitable crops able to generate a comparable income. Nowadays, PVGs are economic unsustainable agrosystems due to the PV shading, but highly profitable for PV energy production. This is the feature that pushes growers and investors to maximize the PV energy production by adopting a high *PV*_*R*_, which improves the overall economic balance of the farm disregarding the yield reduction of the crops. A lower *PV*_*R*_ of the PVG could allow to decrease this disproportion between energy and crop yield (less energy production lead to higher yield), depending on both the crop and the energy tariff. However, in this study the energy price and the feed-in tariff were very high and the crop cannot reach a proportional income, even in the PVG type with *PV*_*R*_ of 25%, in which the PV energy income was two times higher than that of the PV energy. The *PV*_*R*_ level that allows to reach a trade-off between the income from green bean and PV energy was 10%, under the current prices of green bean and electric energy in Italy. This *PV*_*R*_ would allow to achieve a sustainable integration of the PV system and the crop that could contribute equally to the economic income of the greenhouse. However, PVGs with such low *PV*_*R*_ are not spread on the market, given that the high income is easily achievable with the other PVG types.

Under the agronomic perspective, the yield estimation on the other PVG types indicated that green bean is not recommended in PVGs with a *PV*_*R*_ of 50% or more, where the yield reduction is significant (31%), up to 50% inside a PVG with a *PV*_*R*_ of 100% (Table 2). Only the PVGs with a *PV*_*R*_ of 25% can be considered suitable for green bean cultivation, with a yield reduction of 16% on average, compared with a conventional greenhouse. On cycle 3 the PVG type with a *PV*_*R*_ of 60% showed a calculated *P*_*i*_ and *Y*_*t*_ comparable with the experimental value inside the PVG type with *PV*_*R*_ of 50%, due to its higher gutter height (4.5 m instead of 2.5 m of the other types) that let more solar radiation entering the greenhouse from the side walls only when the elevation angle of the sunrays is low, such as in autumn (Cossu et al., 2018). In a PVG with a *PV*_*R*_ of 50% the cumulated solar radiation increases by 4.1% for any additional meter of gutter height. Consequently, increasing the gutter height is a design criteria to adopt in new PVGs with the aim of increasing the availability of solar radiation. The regressions proposed in the study should be considered an estimation to be confirmed with further experimental trials, in order to predict the yield of green bean inside any PVG type.

The E-W orientation is recommended in PVGs because it increases the *I*_*g*_ in winter and decreases it during summer, when less radiation is requested by crops (Sethi, 2009). The *P*_*i*_ decreased gradually on the greenhouse span (N-S direction) from the PL roof to the PV roof (Table 1). Similar trends were already observed on tomato inside the same PVG, where the highest yield was achieved under the PL roof (Cossu et al., 2014). The yield distribution of green bean was decreased by 0.44% per MJ m^–2^ of *P*_*i*_. These data are technically valuable for the estimation of the green bean performance as a function of the actual solar radiation inside any PVG.

### Effect of the Photovoltaic Shading on the Green Bean Quality

The quality in terms of pod weight, length, and caliber was negatively affected by the PV shading (Table 1), since less resources were available, as already observed on shaded common bean (Hadi et al., 2006). This is in agreement with the negative effects on quality (fruit size, firmness, and color) observed on other crops such as tomato and zucchini inside PVGs, even with a *PV*_*R*_ lower than 20% (Minuto et al., 2009; Ureña-Sánchez et al., 2012). In addition, several green bean cultivars grown under shading showed a decrease of pod size, sugars, and malic acid due to the increased respiration in relation to photosynthesis (Vazquez Oderiz et al., 1994; Auerswald et al., 1999; Selan et al., 2014).

The plants under the PV roof were penalized by the light scarcity and suffered from a lower number of leaves, leaf area, and a decrease of LAI and NAR, due to the lower dry weight available for the growth of leaves and beans (Figure 5). The green bean under the PV panels showed a shade-avoidant behavior, concentrating the products of photosynthesis to the stems, which could result in a stem elongation and an increase of plant height. This observation occurred only in cycle 1, with a distribution of dry matter in stems under the PV roof 34% higher than the plants under the PL roof, whereas in cycle 3 the PV shading cast over a higher fraction of the canopy area (especially on rows from 2 to 6), resulting in no statistical difference between PV and PL roof (Figure 6B). The stem elongation is an example of phototropism, in which the plant attempts to surpass the neighbor plants and increase the chance of surviving at a cost of a decrease in the production (Carriedo et al., 2016). The stem elongation is regulated by a combined effect of auxins, gibberellins, and brassinosteroids, that modulate the shade-induced hypocotyl elongation (Yang and Li, 2017; Jiang et al., 2020).

The trend of the photosynthetic rate followed the light distribution inside the PVG (Figure 7). The insufficient light under the PV roof led to average negative net assimilation rates, meaning that respiration can be higher than assimilation even during the day. These physiological parameters were negatively affected also under the PL roof, with an average net assimilation rate of 6.5 μmol CO_2_ m^–2^ s^–1^ during cycle 1, thus, 35% lower than what was observed in unshaded kidney bean, where the average net assimilation of the control crop was constantly higher than 10 μmol CO_2_ m^–2^ s^–1^ (Miyashita et al., 2005).

The plant transpiration rate under the PV roof was 27% lower than that under the PL roof and this difference was noticed during cycle 1, whereas during cycle 3 the transpiration rate was homogenous on the canopy (Figure 7). This aspect highlighted that the shading affects the crop water requirement depending on the position of the plant row inside the PVG and the period of the year. Green beans are sensitive to both water deficit and excess (Sezen et al., 2008; El-Aal et al., 2011; Saleh et al., 2018) and a deficit in specific phenological stages (such as the vegetative growth and flowering) leads to a negative impact on pod yield and quality (Alvino et al., 1988; Boutraa and Sanders, 2001). To avoid this, specific precision fertigation technologies should be applied to differentiate the water and nutrient distribution between the rows, according to the solar radiation distribution on the canopy. These technologies may include the use of electrovalves deployed along each plant row and connected to controllers regulated by the actual irradiance under the conventional (glass or plastic) and PV roof. In fact, the operation of greenhouse controllers for precision irrigation and water management are strongly affected by solar light variations (Kim et al., 2015).

Vegetables grown inside PVGs or agrivoltaic systems show an increase of leaf nitrate content, such as lettuce (Marrou et al., 2013; Sirigu et al., 2019). The EU directives established the limit of nitrate content for greenhouse vegetables, depending on the species (European Commission, 2011). The limit for lettuce is 5,000 mg kg^–1^ and the cultivation inside a PVG with 50% *PV*_*R*_ showed an increase of nitrate content within this limit (Sirigu et al., 2019). However, wild rocket cultivated inside a PVG with 100% *PV*_*R*_ exceeded the EU limit of 7,000 mg kg^–1^, leading to an unmarketable production (Buttaro et al., 2016). According to this, precision fertigation inside PVGs can increase both the nutrient use efficiency and ensure good quality of the fresh produce. The agronomic sustainability and productivity of PVGs can benefit from the application of these technologies, with the aim to reach the optimal agronomic and economic trade-off between energy and food production.

## Conclusion

The green bean cultivation inside a greenhouse with 50% of the area covered with PV panels (PV cover ratio, *PV*_*R*_) was characterized in terms of yield, biomass, and physiological parameters. The linear regression between yield and solar radiation was calculated to estimate the productivity inside other common PVG types with a *PV*_*R*_ ranging from 25 to 100%, based on their light availability. The same approach can be applied to other crops to assess their adaptability inside PVGs. The heterogeneous distribution of the solar radiation negatively affected most of the measured quantity and quality parameters, depending on the position of the plant row under the greenhouse roof. The average green bean yield was 31% lower than in a conventional greenhouse. The number of pods and their average weight and length decreased under the PV panels, as well as LAI, plant dry weight, net assimilation, and transpiration rate. In particular, the net assimilation under the PV panels was occasionally negative also during the day. On the other hand, these plants showed a higher RUE by increasing their SLA, LAR, and relocating resources to the stems. The income from PV energy production per square meter resulted four times higher than that of the green bean yield, showing that a PV_*R*_ around or higher than 50% is not sustainable to ensure a good income from crop production. On the other hand, a *PV*_*R*_ equal or lower than 10% can potentially result in balancing the two incomes, leading to a sustainable integration of the PV energy on the greenhouse crop. Compared with a conventional greenhouse, the average estimated yield of the other PVG types considered in the study showed a reduction ranging from 16 to 52% inside the 25% gable roof type and the 100% mono-pitched roof type, respectively. The green bean yield increased averagely by 0.44% per each additional 1% cumulated PAR and this experimental and technical data is valuable for estimating the crop productivity inside PVGs. The transpiration rate decreased by 27% under the PV roof, compared with that of the PL roof. This latter finding suggested that the water and nutrient requirements changed between the plant rows, and further studies are needed to optimize the water management inside PVGs through precision irrigation technologies.

## Data Availability Statement

The raw data supporting the conclusions of this article will be made available by the authors, without undue reservation.

## Author Contributions

MC conceptualized and designed the work, handled the data analysis, software, visualization, and wrote, reviewed, and edited the original manuscript. AS was in charge of the data collection, funding acquisition, investigation, methodology, project administration, resources, and reviewed, edited, and wrote the manuscript. PD also handled the investigation, reviewed, edited, and wrote the manuscript. RF and GU handled the data collection and investigation. GC performed data analysis. LL conceptualized and designed the work and was also in charge of the funding acquisition and supervision. All authors contributed to the article and approved the submitted version.

## Conflict of Interest

The authors declare that the research was conducted in the absence of any commercial or financial relationships that could be construed as a potential conflict of interest.

## Abbreviations

*A*: Leaf area (cm^2^)
BOS: Balance of system
CV: Coefficient of variation (%)
EUE: Energy use efficiency of the PV system (kWh MJ^–1^ m^–2^)
*f*_*P*_: Fraction of PAR radiation to *I*_*g*_ inside the PVG (dimensionless)
*f*_*PL*_: Fraction of PAR to *I*_*g*_ under the PL roof (dimensionless)
*f*_*PV*_: Fraction of PAR to *I*_*g*_ under the PV roof (dimensionless)
*F*_*sh*_: Percentage of the greenhouse area shaded by the PV panels (%)
*G*_*GR*_: Percentage of PAR inside the PVG compared to a conventional greenhouse (%)
*I*_*g*_: Global irradiance (W m^–2^)
LAI: Leaf area index (dimensionless)
LAR: Leaf area ratio (cm^2^ g^–1^)
*n*: Crop cycle duration (d)
NAR: Net assimilation rate of the leaf (g m^–2^)
PAR: Photosynthetic active radiation (W m^–2^)
*P*_*i*_: Cumulated PAR inside the PVG (MJ m^–2^)
PL: Plastic
*P*_*o*_: External cumulated PAR (MJ m^–2^)
PV: Photovoltaic
PVG: Photovoltaic greenhouse
*PV*_*R*_: Photovoltaic cover ratio (%)
*RH*_*a*_: Average relative humidity (%)
*RUE*_*F*_: Radiation use efficiency on fresh weight (g MJ^–1^)
SLA: Specific leaf area (cm^2^ g^–1^)
*T*_*b*_: Base temperature (°C)
*T*_*m*_: Daily mean temperature (°C)
*T*_*s*_: Daily temperature sum (° C days)
*W*_*d*_: Plant dry weight (g m^–2^)
*W*_*f*_: Plant fresh weight (g m^–2^)
*W*_*l*_: Leaf dry weight (g m^–2^)
*y*_*c*_: 1% rule coefficient (%)
*Y*_*t*_: Total fresh yield (kg m^–2^)
δδ: Sun elevation angle (°)
ηη_*m*_: PV module efficiency (%)
ηη_*PV*_: PV system overall efficiency (%)
τ: Average greenhouse transmissivity (dimensionless).
